# Is handgrip strength normalized to body weight a useful tool to identify dynapenia and functional incapacity in post-menopausal women?

**DOI:** 10.1590/bjpt-rbf.2014.0184

**Published:** 2016-09-22

**Authors:** Maude Dulac, Guy El Hajj Boutros, Charlotte Pion, Sébastien Barbat-Artigas, Gilles Gouspillou, Mylène Aubertin-Leheudre

**Affiliations:** 1 Faculty of Science, Department of Exercise Science, University of Quebec at Montreal, Montreal, Quebec, Canada; 2 Groupe de Recherche en Activité Physique Adapté – GRAPA, Department of Exercise Science, University of Quebec at Montreal, Montreal, Quebec, Canada; 3 Research Centre of the Montreal Geriatric, McGill University, Montreal, Quebec, Canada; 4 Faculty of Biology, University of Quebec at Montreal, Montreal, Quebec, Canada; 5 Faculty of Biology, Department of Neurosciences, University of Montreal, Montreal, Quebec, Canada

**Keywords:** aging, muscle strength, physical performance

## Abstract

**Objective:**

To investigate whether handgrip strength normalized to body weight could be a useful clinical tool to identify dynapenia and assess functional capacity in post-menopausal women.

**Method:**

A total of 136 postmenopausal women were recruited. Body composition (Dual Energy X-ray Absorptiometry [DEXA], Bio-electrical Impedence Analysis [BIA]), grip strength (dynamometer) and functional capacity (senior fitness tests) were evaluated. Dynapenia was established according to a handgrip strength index (handgrip strength divided by body weight (BW) in Kg/KgBW) obtained from a reference population of young women: Type I dynapenic (<0.44 kg/KgBW) and type II dynapenic (<0.35 kg/KgBW).

**Results:**

The results show a positive correlation between handgrip strength index (in kg/KgBW) and alternate-step test (r=0.30, p<0.001), chair-stand test (r=0.25, p<0.005) and one-leg stance test (r=0.335, p<0.001). The results also showed a significant difference in non-dynapenic compared to type I dynapenic and type II dynapenic for the chair-stand test (Non-dynapenic: 12.0±3.0; Type I: 11.7±2.5; Type II: 10.3±3.0) (p=0.037 and p=0.005, respectively) and the one-leg stance test (Non-dynapenic: 54.2±14.2; Type I: 43.8±21.4; Type II: 35.0±21.8) (p=0.030 and p=0.004, respectively). Finally, a significant difference was observed between type II dynapenic and non-dynapenic for the chair-stand test (p=0.032), but not with type I dynapenic.

**Conclusion:**

The results showed that handgrip strength was positively correlated with functional capacity. In addition, non-dynapenic women displayed a better functional status when compared to type I and type II dynapenic women. Thus, the determination of the handgrip strength thresholds could be an accessible and affordable clinical tool to identify people at risk of autonomy loss.

## BULLET POINTS

- Aging is associated with loss of autonomy, increased risk of falls, and mortality.
- Handgrip strength is positively correlated with functional capacity.
- Handgrip strength thresholds could be an accessible/affordable clinical tool.

## Introduction

Aging is associated with loss of autonomy, increased risk of falls, fractures and mortality^1^ ^-^ ^3^. The relation between independence and functional capacities has been observed in many studies^1^ ^,^ ^3^ ^-^ ^5^. However, the question that remains is what kind of clinical criteria could be used to assess the level of functional capacity in individuals. Losses of muscle mass and muscle strength have been identified as keys components in the loss of functional capacity with aging. Recently, Clark and Manini^6^ proposed dividing loss of muscle mass (sarcopenia) and loss of muscle strength (dynapenia) to explain functional disabilities. There is now evidence to suggest that loss of strength, or dynapenia, is a more important factor than the decline in muscle mass^7^ and that muscle quality (i.e. muscle strength/muscle mass)^2^ prevails over sarcopenia and dynapenia alone. Indeed, Newman et al.^8^, found that muscle strength (i.e. leg extension strength with an isokinetic dynamometer; grip strength with an isokinetic dynamometer) and mortality were strongly correlated, while the correlation between muscle mass and mortality was relatively weak^8^. It also has been demonstrated that low levels of lower-limb and upper-limb muscle strength are associated with greater mortality in the elderly^8^. Moreover, Bouchard et al.^9^ found that quadriceps strength (i.e. leg extension with an isokinetic dynamometer) and fat mass were better predictors of physical function than muscle mass in older adults^9^. Furthermore, Choquette et al.^10^ also found that elderly in the lowest and middle tertile of 1) handgrip strength / body mass index or 2) quadriceps strength / body weight were more likely to have a lower mobility score^10^. Barbat-Artigas et al.^11^, who observed that handgrip strength/body weight was a better clinical tool than handgrip/body mass index in predicting functional incapacities, have confirmed these results^11^. Finally, Visser and Schaap^12^ and Barbat-Artigas et al.^13^ concluded that to prevent functional decline, falls, and early mortality in older men and women, a major focus on maintaining or increasing muscle strength instead of muscle size was warranted.

However, one of the major challenges in the field is to adequately standardize the identification of dynapenic individuals. In fact, some studies^14^ ^,^ ^15^ use different criteria to identify loss of muscle strength, which has rendered findings and/or conclusions difficult to interpret or generalized.

In the context of clinical research and medical assessment, handgrip strength would be an easier tool of measure. Recently, Alley et al.^16^ proposed an absolute clinical cut-point with handgrip divided by body weight to identify populations who might benefit from interventions to improve muscle strength and function. However, it was important to note that Choquette et al.^10^ and Barbat-Artigas et al.^13^ showed that handgrip/BW is a better clinical predictor of functional impairments than handgrip alone in older men and women. Thus, it is important to define a clinically standardized cut-point to identify dynapenic individuals using handgrip/body weight, which could lead to the efficient identification of patients at risk of functional incapacities.

Accordingly, the aims of the present study were (1) to identify functional differences between type I and type II dynapenic postmenopausal women based on handgrip strength normalized by total body weight values obtained from a reference population of young women and (2) to define whether this potential clinical index was associated with functional capacity in postmenopausal women.

## Method

### Participants

A total of 136 non-frail postmenopausal women aged from 50 to 75 years (means: 61±6 yrs.) were recruited using advertisements in local newspapers and in the community. It should be noted that none of the participants were identified as frail, which was based on the criteria of Fried et al.^17^. To be included in the study, women had to meet the following criteria: no major physical incapacities (i.e. Classified as autonomous related to the 18 activities of daily living), no history of cardiovascular diseases or diabetes, no medication that could influence metabolism (except hormonal therapy), non-smoker, moderate drinker (less than 2 drinks/day), a body mass index (BMI) of 18.5 to 35 kg/m^2^, stable weight (±2 kg; self reported) for the last 6 months, an absence of menses for the past 12 months, and non-physically active (less than 2 hours a week of self-reported structured exercise). All procedures were approved by the Ethics Committee of the Department of Exercise Science of the Université du Québec à Montréal, Montréal, Québec, Canada (2010-A-109987). All participants were fully informed of the nature, goal, procedures and risks of the study, and gave their informed consent.

### Design

A phone interview was conducted to screen for the aforementioned inclusion criteria. After screening, women were invited for a visit to the Department of Exercise Science at the Université du Québec à Montréal. After their arrival, body composition was measured in a fasting state; thereafter muscle strength and functional capacities were assessed.

### Body composition

*Anthropometric Measurements*: Body weight (BW) was determined using an electronic scale (Omron HBF-500CAN, USA). Height was measured using stadiometer (Seca, USA) affixed to the wall. BMI was calculated using the following formula: BW kg/height^2^ (m).

*Body composition*: Fat mass (FM) and lean body mass (LBM) percentages were measured by bio-electrical impedancemetry analysis (BIA; Omron HBF-500CAN, USA) based on the Fricke model^18^. The measurements were taken in the early morning after an overnight fast (food and liquid). Whole-body BIA measurements were taken with the participant standing upright, barefoot on the scale, arms stretched out in a 90° abduction position, holding the handle of the device. In our laboratory, the coefficients of variation for repeated measures of body composition in 10 young women was 0.5% for fat mass and 0.5% for lean body mass.

*Skeletal Muscle Mass*: Body resistance was measured by BIA. The measurements were taken in the early morning after an overnight fast (i.e. food and liquid). In our laboratory, the coefficient of variation for repeated measures of body resistance in 10 young women was 1%. Skeletal muscle mass (SM) was estimated using the validated Equation 1 of Janssen et al.^5^:

*Skeletal muscle mass (kg)* = (Height (cm^2^)/*Resistance (Ω)* × 0.401) + (sex (female = 0) × 3.825) + (age (years) × 0.071) + 5.102 (1)

### Muscle strength

*Handgrip strength*: Maximum voluntary handgrip strength was measured with a hand dynamometer with adjustable grip (Hand Dynamometer, Lafayette Instrument, USA). This method has been shown to be reliable^19^. Participants were standing upright with the arm along the side of the body without any elbow flexion, with the palm of the hand facing the thigh. Participants were instructed to apply as much handgrip pressure as possible for at least 4 seconds, performing the test with the right and left hands in turn. They performed three trials for each hand. The maximum score for each hand was recorded.

Then, muscle strength was expressed as absolute muscle strength divided by body weight (kg/BW). Muscle strength corresponded to the maximal handgrip strength the participant was able to produce. Handgrip strength was used because of the accessibility and viability of this measure^8^. Muscle strength was also normalized by body weight taking into account the higher functional incapacity risk in obese individuals^10^. In attempt to establish a clinical criterion for the diagnosis of the risk of functional capacity, and the sub-sequent loss of autonomy^20^, observed in elderly individuals, handgrip strength divided by body weight was chosen.

*Dynapenia criteria*: Dynapenia was defined using a reference population as previously described by Barbat-Artigas et al.^21^. Thus, Type I dynapenia was defined as a muscle strength index value of 1 to 2 standard deviations below the average muscle strength index of our reference population, whereas type II dynapenia represented a muscle strength index of 2 standard deviations or more below the same value. Thus, women were considered non-dynapenic with a muscle strength index value of >0.44 kg/BW and type I and type II dynapenic respectively with a muscle strength index value of <0.44 kg/BW and <0.35 kg/BW.

### Functional capacities

Functional capacities were assessed using the following three tests^22^: (1) The **alternate-step test,** conducted by alternatively placing the whole left and right feet as fast as possible onto a step for a 20-second period; (2) **The chair-stand test,** conducted with the participant having the arms crossed at the wrists and held close to the chest, standing up straight from a chair without arm rests, then completely sitting back down, as fast as possible for a 20-second period; and (3) **The one-leg stance test,** conducted by standing on one leg for as long as possible with the arms along the side of the body. The test was interrupted after 60 seconds or if the participant touched the floor with the swing leg. The measures were performed to the right and left legs, with the eyes opened. The best score was recorded.

### Physical activity level

Because the self-reported initial criteria to be included in this study is not accurate enough to identify physical activity level among elderly people, this important factor was controlled according to a more accurate method used to assess physical level. Recreational exercise habits (i.e. planned, structured, and repetitive bodily movement done to improve or maintain one or more components of physical fitness) had been identified using a structured interview conducted by a trained kinesiologist using a form comprising activities and blank spaces to allow recording of unlisted activities. This assessment method was chosen over more objective methods (e.g., accelerometer) because it allowed the assessor to cover and identify all exercises (e.g., resistance training, walking, swimming, martial arts, yoga), it allowed the assessor to collect accurate information for each activity, and it was clinically accessible. Participants were asked to specify the exercise time (in min/week) for each activity in which they were currently engaged and that these activities had been practiced (in months). Activities were then categorized into three main categories: resistance, aerobic, and body and mind exercises. Based on this information, the weekly exercise time was calculated as well as the average exercise duration in total or by category of activity.

### Statistical analysis

Results are presented as means ± SD. Based on the literature^2^ and according to our population and our intervention; a sample size of 20 participants in each group was needed for each group for a power of 80% and an alpha error of 0.05.

Normality was verified using the Kurtosis-test. Pearson correlations were performed between muscle strength (i.e. handgrip in kg/BW) and functional capacity tests (i.e. alternate-step test, chair-stand test, one-leg stance test). Non-parametric tests were performed to compare physical characteristics and functional capacity among participants according to dynapenia criteria because some of the groups were composed with less than 25 individuals even if the variables were considered normally distributed. The non-parametric tests used were Kruskall Wallis test with Mann Whitney post hoc and Bonferroni adjustment. P values ≤0.05 were considered statistically significant. Analyses were performed using SPSS 17.0 software (Chicago, IL). For correlation interpretation, the *r* values were interpreted using the following guidelines: 0.00 to 0.19 = none to slight, 0.20 to 0.39 = low, 0.40 to 0.69 = modest, 0.70 to 0.89 = high, and .90 to 1.00 = very high^23^.

## Results

**Figure 1:**
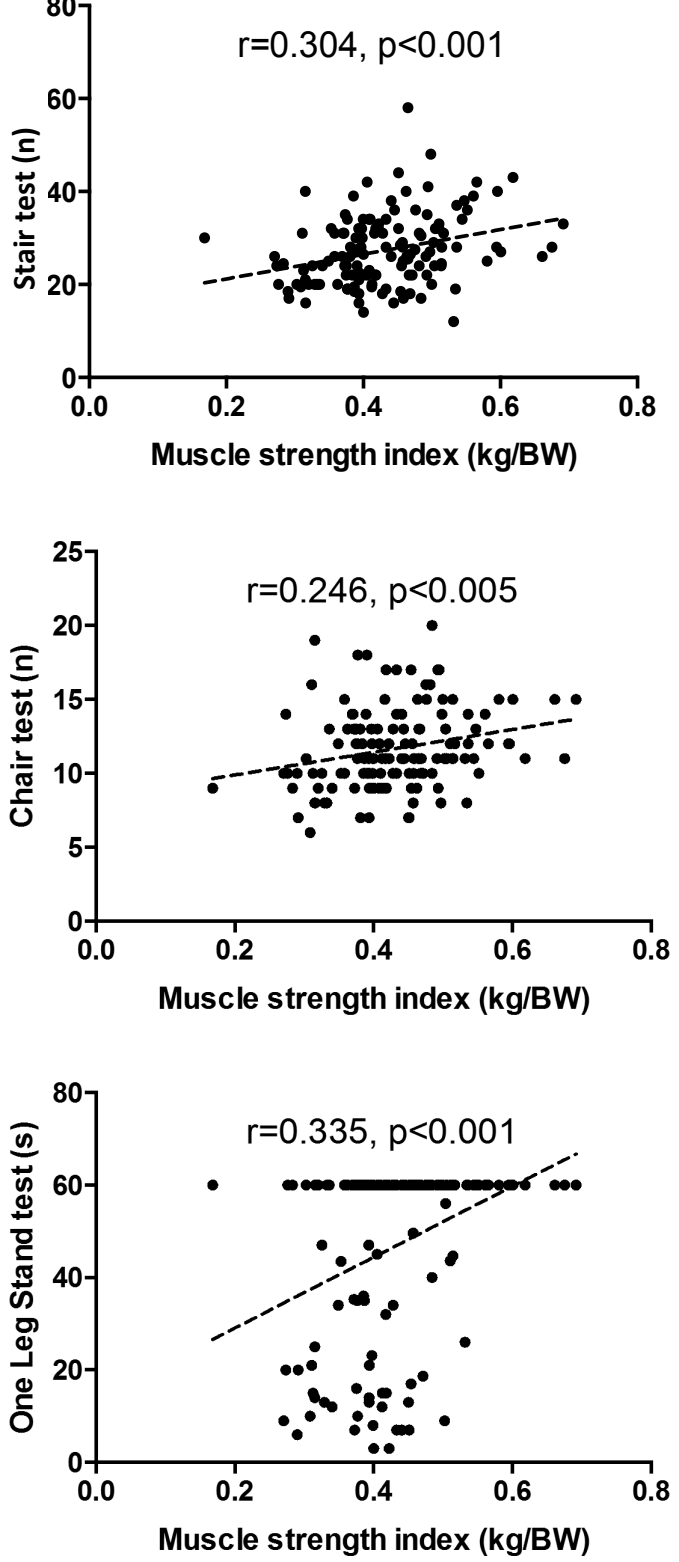
Correlation between muscle strength index (kg/BW) and functional capacities in post-menopausal women (n=136).

A positive correlation was observed between the handgrip strength index (in kg/BW) and the alternate-step test (r=0.30, p<0.001), chair-stand test (r=0.25, p<0.005) and one-leg stance test (r=0.34, p<0.001) (Figure 1).

Table 1 shows the physical characteristics of non-dynapenic, type I dynapenic and type II dynapenic subjects. Significant differences between non-dynapenic and type II dynapenic were observed for age and physical activity level. Significant differences were also found between all groups for BMI, handgrip strength and fat mass. However, no difference were observed between groups for lean body mass.

**Table 1:** Physical characteristics of the post-menopausal women according to dynapenia.

	Non-dynapenic	Type I dynapenic	Type II dynapenic
	Mean±SD	Mean±SD	Mean±SD
	n=58	n=56	n=22
Age (years)	60.2±6.7†	62.1±6.6	64.0±6.2
Height (cm)	160.87±5.86	159.43±7.24	161.97±7.25
Body weight (kg)	60.81±7.62‡ †	66.52±10.36*	82.15±23.72
BMI (kg/m^2^)	23.5±2.8‡ †	26.2±3.8*	31.1±7.5
Lean body mass (kg)	20.7±5.6	21.3±7.5	23.4±14.6
Handgrip Strength (kg/KgBW)	0.51±0.06‡ †	0.40±0.02*	0.31±0.04
Fat mass (%)	33.3±5.7‡ †	36.6±6.1*	43.1±6.3
Physical activity level (min/day)	37.0±20.7†	27.7±21.7	16.3±12.2

Values are expressed as Mean±SD. BMI: Body mass index;

‡ significant difference between non-dynapenic and type I dynapenic;

* significant difference between type I dynapenic and type II dynapenic;

† significant difference between non-dynapenic and type II dynapenic.

Table 2 shows functional capacity characteristics of non-dynapenic, type I dynapenic and type II dynapenic subjects. A significant difference was observed between dynapenic (type I and type II) and non-dynapenic subjects for the alternate-step test (p=0.002), chair-stand test (p=0.050) and one-leg stance test (p<0.001). A significant difference was also observed for the alternate-step test and one-leg stance test for non-dynapenic compared to type I and type II dynapenic (alternate-step: p=0.037 and p=0.005; one-leg stance: p=0.030 and p=0.004 respectively). No significant difference was observed for the chair-stance test between non-dynapenic and type I dynapenic and between type I and type II dynapenic. In contrast, a significant difference was observed between type II dynapenic subjects and non-dynapenic individuals for the chair-stand test (p=0.032). Finally, even when controlling for physical activity level, all of the previously mentioned differences remained significant (data not shown).

**Table 2:** Difference in functional capacities using 3 functional tests between non-dynapenic, type I dynapenic and type II dynapenic post-menopausal women.

	Muscle strength index (kg/KgBW)			
	Non-dynapenic	Type I dynapenic	Type II dynapenic	*p*
	n=58	n=56	n=22	
Alternate-step test	29.8±8.5‡ †	26.3±6.5	23.4±5.7	0.002
Chair-stand test	12.0±3.0†	11.7±2.5	10.3±3.0	0.050
One-leg stance test	54.2±14.2‡ †	43.8±21.4	35.0±21.8	<0.001

‡ significant difference between non-dynapenic and type I dynapenic;

† significant difference between non-dynapenic and type II dynapenic.

## Discussion

The aim of this study was to define whether handgrip strength/body weight could be considered as a useful clinical criterion for identifying dynapenia and assessing functional capacity in post-menopausal women. The findings of this study showed that handgrip strength divided per body weight was positively and significantly correlated with functional capacity in postmenopausal women. Furthermore, when used to identify dynapenic versus non-dynapenic individuals, the handgrip strength normalized for total body weight identified non-dynapenic women having significantly superior functional capacities values as compared to type I and type II dynapenic on women. These results are in line with previous studies, which reported a relationship between muscle strength and functional capacity. However, the results of these previous studies used different methods for measuring (e.g. leg press, leg extension^10^ ^,^ ^15^ ^,^ ^20^ and expressing muscle strength (handgrip in absolute^20^, handgrip/BMI^13^). Thus, it was deemed useful to develop and propose a more practical and clinical method. That is, handgrip normalized by BW could display a clinically relevant sensitivity to unmask the relationship between muscle strength and functional capacities while offering a high degree of simplicity. The strength of this relationship was reinforced by the fact that the relationship was present even in a homogenous population of postmenopausal women.

It should be noted that lean body mass was not different between groups, which was consistent with the findings of Bouchard et al.^9^, Barbat-Artigas et al.^11^ and the conclusions of Visser and Schaap^12^, which strengthen the idea that the decline in muscle strength might play a more important role than the decline in muscle mass in the deterioration of functional capacity. In addition, in the present study, even within a population of similar lean body mass content, the relationship between handgrip strength and functional capacity existed.

The present study had several limitations. First, a cross-sectional approach was used, which did not allow us to conclude to any causal associations between muscle strength and functional capacity in the cohort tested. Second, correlation was used which cannot be used to infer causality. Finally, being unable to control for a number of factors such as the hormonal changes or the time spent in hormonal therapy for each participant became a limitation to the present study. Indeed, these factors could influence the pace of the loss of muscle strength.

In conclusion, the present study indicated that the handgrip strength normalized per body weight could be associated with functional capacities in postmenopausal women, despite similar muscle mass. As such, the handgrip strength normalized per body weight appeared as an interesting dynapenic index that could prove useful for clinicians in the identification of individuals at risk of mobility impairment. Furthermore, the cut-point values that were used to identify dynapenic subjects in the present study may be used by other studies with a larger sample size as the cornerstone for the development of a standardized cut-point for the identification of functional impairment. More studies with more heterogeneous groups and larger sample sizes are desirable to confirm the present study’s findings, these studies may be required to identify precise functional cut points from this index and also to improve the association between muscle strength and functional capacity.
